# The Evolution of Current Concept of the Reconstructive Ladder in Plastic Surgery: The Emerging Role of Translational Medicine

**DOI:** 10.3390/cells12212567

**Published:** 2023-11-03

**Authors:** Francesco De Francesco, Nicola Zingaretti, Pier Camillo Parodi, Michele Riccio

**Affiliations:** 1 Department of Reconstructive Surgery and Hand Surgery, University Hospital (AOU Ospedali Riuniti di Ancona), Via Conca 71, Torrette di Ancona, 60123 Ancona, Italy; michele.riccio@ospedaliriuniti.marche.it; 2 Department of Medical Area (DAME), Clinic of Plastic and Reconstructive Surgery, Academic Hospital of Udine, University of Udine, 33100 Udine, Italy; zingarettin@gmail.com (N.Z.); piercamillo.parodi@uniud.it (P.C.P.)

**Keywords:** reconstructive ladder, translational medicine, adipose tissue, reconstructive translational ladder, exosomes, 3D bioprinting, dermal template, reconstructive plastic surgery

## Abstract

Plastic surgeons have used the reconstructive ladder for many decades as a standard directory for complex trauma reconstruction with the goal of repairing body structures and restoring functionality. This consists of different surgical maneuvers, such as secondary intention and direct tissue closure, as well as more complex methods such as local tissue transfer and free flap. The reconstructive ladder represents widely known options achievable for tissue reconstruction and wound closure that puts at the bottom rung the simplest methods of reconstruction and strengthens the complexity by moving upward. Regenerative medicine and surgery constitute a quickly spreading area of translational research that can be employed by minimally invasive surgical strategies, with the aim of regenerating cells and tissues in vivo in order to reestablish normal function through the intrinsic potential of cells, in combination with biomaterials and appropriate biochemical stimuli. These translational procedures have the aim of creating an appropriate microenvironment capable of supporting the physiological cellular function to generate the desired cells or tissues and to generate parenchymal, stromal, and vascular components on demand, and above all to produce intelligent materials capable of determining the fate of cells. Smart technologies have been grown that give extra “rungs” on the classic reconstructive ladder to integrate a more holistic, patient-based approach with improved outcomes. This commentary presents the evolution of the traditional concept of the reconstructive ladder in the field of plastic surgery into a new course with the aim of achieving excellent results for soft tissue reconstruction by applying innovative technologies and biologically active molecules for a wide range of surgical diseases.

## 1. Reconstructive Ladder

Many surgeries carry out a demolitive and reconstructive activity but those that are truly “reconstructive” are distinguished based on their ability to provide not only repair of the defect but also on their ability to provide a restoration of the function of the damaged organ. Historically, the term reconstructive is above all the prerogative of that surgical specialty that is able to reconstruct a defect through the use of new parts or a local reorganization of the defect itself [1]. In particular, the “reconstructive ladder” represents the guideline of all surgeons in this discipline [2,3]. A guiding principle establishes that wound repair should begin with a simple method, keeping in mind that, in certain clinical situations, more advanced techniques will be necessary. In this regard, the reconstructive ladder represents the wide spectrum of reconstructive possibilities available to the plastic surgeon [4], from the simplest to the most complex option. The strategy of choosing the best option (grafts, flaps, and microsurgery) for substance loss requires a deep understanding of tissue biology, wound healing physiology, and anatomy. Despite this, the surgeon’s clinical intuition regarding not only the factors that affect wound healing; his own surgical skill is also important. The principal aim of reconstruction consists of “wound coverage”, “infection control”, “anatomical replacement”, “functional maintenance”, and “aesthetic amelioration”. The first two are fundamental for the patient’s problem resolution. The last two are important for the improvement of the patient’s life quality. Moreover, anatomical replacement is related to functional and aesthetic outcomes for the restoration of the patient’s defect. Obviously, based on the site defect, functional loss, and reconstructive difficulty, the surgeon chooses the best reconstructive strategy, based on the reconstructive ladder and on their skill. In recent years, the principles of the reconstructive ladder had become a truly universal dogma, whereby the simplest technique was taken into consideration without considering the final functional result and the transition to the subsequent rungs was used only when there was no other solution [5]. This dogma has obviously been overcome by reconstructive surgeons, by introducing the concept of the “reconstructive elevator” [6], as it allows you to jump from one step to another in a creative way and above all according to the patient’s needs in order to achieve the desired result. This school of thought was born, principally, with the advent of microsurgery [7]. This school also has its disadvantages, in that the principles may be attractive for wound healing, but the lift leads the reconstructive surgeon to use, inevitably, more complex techniques such as free flaps. It is not the technique that solves the problem, but the problem that calls for a particular type of technique depending on the site, function, aesthetic considerations, and morbidity. In this regard, in recent years different opinions have developed regarding the reconstructive possibilities [8,9,10]. This is certainly due to the implementation of surgical techniques becoming more and more sophisticated and therefore the authors have attempted to improve the different “ladders” by adding rungs for an application more suitable for different scenarios. Even if the reconstructive ladder sustained moderate modification over time, the fundamental view of reconstructive procedures graded by difficulty has been maintained and been spread in different theories. The ever-increasing complexity of injuries has led to greater interaction between basic sciences and surgery [11,12,13] to create new and innovative techniques for tissue regeneration. Farid and colleagues [14] performed a critical review of the concept of the reconstructive stair and elevator opening the doors for the use of new technologies. Translational medicine is made up of a multidisciplinary team [15,16] that applies modern regenerative-medicine techniques to complex traumas with the aim of improving the results expected by the patient.

## 2. Translational Reconstructive Ladder

Translational medicine (TM) can be described as the interdisciplinary use of biomedical investigation for the enhancement of different disease conditions; in particular, the European Society for Translational Medicine (ESTM) has established that translational medicine is supported by three fundamental pillars (bench, bedside and community) with the aim of improving the health of society through the development of new therapies [17]. Despite encouraging preclinical and clinical results, the high degree of heterogeneity in MSC stemness and differentiation potential represents a challenge for reproducibility and therapeutic standardization. MSC heterogeneity can occur at multiple levels, depending on the donor, tissue source, isolated cell subset, and manipulation techniques [18]. A promising route to improving the efficiency of MSCs is to select and isolate specific sub-populations, using cell sorting based on specific markers. Likewise, there is no scaffold that is suitable for every type of tissue to regenerate. Today, scaffold designs are controlled at the nano- or micro-scale and are based on the property of the extracellular matrix (ECM). The ECM provides a variety of physical, chemical, and biological signals that influence cell growth and proliferation. At the same time, the design of smart constructs must take into account the specific characteristics of the tissue and must be “physiologically relevant”, considering the specific anatomical and functional properties of the tissue [19,20]. To improve the “physiological relevance” of engineered constructs, it is important to understand the biological context, such as ECM, vasculature, and cell type, as well as the different chemical, physical, mechanical, and spatial signals; however, timescales should also be considered [21]. In the last decade, the increasingly strong interaction between translational medicine and clinical practice has become the cornerstone of the new biomedicine, ushering in a real revolution in modern science. This integration, which embraces all medical specialties but finds its maximum application in reconstructive surgery, allows the new technologies to offer new opportunities in the treatment of diseases to the point of currently having a prominent role in the community [22,23]. The translation of regenerative approaches into clinical practice is limited by the strict legal regulation of in vitro expanded cells and the risks associated with substantial manipulations. Therefore, micrografts created directly in the operating room (OR) with minimal cell manipulation appear extremely promising, and has demonstrated its efficacy in recent clinical trials [24,25,26].

**Figure 1:**
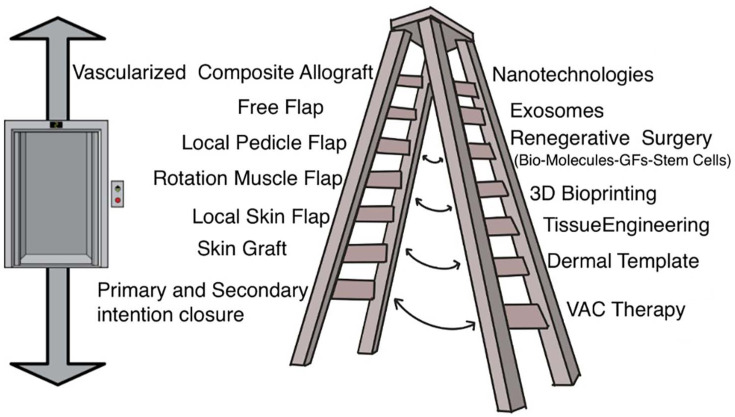
The figure shows the “translational reconstructive ladder”, incorporating the classic reconstructive ladder (**left**) and the translational clinical procedures (**right**) as a booklet scale. The reconstructive elevator can be applied to both sections of the ladder and, as well, the surgeon can switch from one side to the other of the two sections depending on the patient’s clinical condition.

Smart technologies have been developed that provide extra rungs on the reconstructive ladder to integrate the principles of translational medicine into a global approach, in which the center is not only represented by the patient but also by the community. In this regard, the traditional reconstructive scale, although providing excellent outcomes, does not take into account the economic and healthcare problems linked to the treatment process [27,28]. The “translational reconstructive ladder” paradigm represents a conjunction of regenerative-medicine therapies with traditional reconstructive approaches (Figure 1). This original paradigm can be applied: (i) to large wounds with extensive soft tissue losses, to obtain a better and definitive closure; (ii) pain therapy; (iii) inflammatory and chronic pathologies; (iv) vascular pathologies; (v) post-cancer reconstruction; and many others. Moreover, translational medicine involves the use of cell- and tissue-engineering scaffolds, decellularized extracellular matrix, wearable medical sensors, micro- and nano-medicine, 3D bioprinting, biologically inspired engineering, organ chips, and bioelectronics. The advent of microsurgery has brought important improvements in autologous reconstructive options [29,30,31,32]; however, the morbidity of the donor site and the scarring outcomes still remain prevalent [33,34]. Reconstructive plastic surgery aims to provide vital tissue and, above all, replacing like tissue with like tissue, respecting anatomical zones, to restore a wide range of defects. Plastic surgeons, in this regard, are in a favorable position due to the versatility deriving from the use of all human tissues including the skin, fat, nerves, muscles, bones and cartilage, but above all for being intrinsically involved in the research and development of engineered tissues in the laboratory and in their clinical use [35]. Thanks to the combination of microsurgery, composite vascularized allografts [36,37] and nanotechnologies [38], biomaterials [39], and 3D printing [40], the weapons in the plastic surgeon’s baggage are notable both for the reconstruction of large defects and for the reduction of scarring in donor sites, with consequent reduction of the associated hospital care (Figure 1).

### 2.1. Autologous Adipose Tissue Grafting and Adipose Stem Cells (ASCs)

The most classic clinical approach, through translational medicine, is the use of tissue-derived cells seeded on scaffolds [41]. The preferred choice is the use of autologous cells, to avoid immunogenicity problems. The cells used can be of adult origin, and therefore completely differentiated [42], or multipotent progenitor cells with low proliferative potential and their differentiation commitment, and finally stem cells capable of proliferating through multiple generations and differentiating into a variety of cell types [43]. Adult stem cells have been isolated from various tissues, such as bone marrow [44,45], epithelial tissue [46], umbilical cord tissue [47,48], and adipose tissue [49,50,51,52]. Plastic surgeons are very familiar with the use of adipose tissue to restore subcutaneous tissue associated with scar contractures or body contour deformities. This procedure is called lipofilling or fat grafting [53]. There are currently numerous procedures to try to obtain the best purified fat through various mechanical systems for adipose tissue concentration [54,55]. The aim is to preserve the components of the lipoaspirate, such as adipocytes, pre-adipocytes, and stroma. Furthermore, within the adipose tissue it was possible to isolate a portion of stem cells that was called the stromal vascular fraction [56]. The stromal vascular fraction (SVF) is represented by diversified cell populations, such as stem cells, preadipocytes, endothelial cells, pericytes, T cells, and M2 macrophages [57], and capable of multipotency differentiation [58,59,60]. Adipose tissue solutions contain many sources of growth factors, cytokines, adipokines, and transcriptional factors, which together produce secretomes. These acellular secretomes have extensive biological activity [61]. In this regard, the therapeutic capacity of MSCs can be increased by gene modification to force the expression of paracrine activity. In a recent review [62], the authors showed how therapies based on engineered MSCs have been used for the treatment of acute graft-versus-host, limb ischemia, and perianal fistulas in Crohn’s disease. In these studies, genetically modified or engineered MSCs were used in which the production of specific paracrine factors, necessary for a particular pathological condition, was forced. By exploiting the paracrine potential of ASCs secretomes, they act directly at the site of inoculation, activating a clinical response with an enormously increased regenerative potential [63]. Among all the products derived from adipose tissue, currently, through modern regenerative-medicine techniques and based on the current legislation, it is possible to use above all these biological peptides, such as exosomes [64,65]. Exosomes are an innovative boundary of intercellular connection managing the cells’ biological action such as immunomodulation and anti-inflammatory properties [66]. Exosomes derived from adipose stem cells are a significant unit liberating by SVF and have many biological activeness [67]. There is evidence of regenerative potential in dermatological disorders such as scars [68], wrinkles [69], pigmentation [70]; in plastic surgery as chronic wounds [71,72], in the orthopedic field such as small joints [73,74,75], and tendons [76,77]. Exosomes have acquired much consideration because they are substantial paracrine mediators providing tissue regeneration. Furthermore, exosomes derived from adipose tissue have the ability to encapsulate various types of bioactive molecules and therefore have great application potential in tissue regeneration [78,79]. Although various findings highlight the significant role of adipose tissue-derived exosomes for tissue regeneration in the field of reconstructive plastic surgery, adequate application in clinical practice is currently lacking. In this regard, the link provided by translational medicine, and in particular by the translational scale, can provide an increasingly important role for these mediators to maximize the therapeutic effect in a large variety of pathologies.

### 2.2. ECM Scaffold and Dermal Regeneration Template (DRT)

A suitable scaffold is essential to any tissue-engineering strategy. The scaffold supplies a structure for the cell growth, permitting cells to attach, proliferate, and differentiate, at the same time as performing cellular running into a workable 3D network [80]. Moreover, the preferable scaffold should be biomimetic, biodegradable, biocompatible, and non-immunogenic [81] but also should have appropriate mechanical strength and optimal micropores [82]. Finally, the scaffold should be suitable for clinical grade sterilization and industrial production. A cell-scaffold construct can be attempted in vitro in a bioreactor or in vivo by implanting scaffold with autologous cells into the body. Research on biomaterials aims to design “functionalized” or “smart” scaffolds that, on the one hand, incorporate cells on the surface in order to create a new tissue and on the other hand release biomolecules, growth factors, or antibiotics over time capable of prolonging their activities [83]. The dermal regeneration template (DRT) is a three-dimensional bioactive scaffold that actually constitutes the regenerative-medicine applications in the reconstructive fields [84,85]. These DRT materials are capable of supporting tissue regeneration and remodeling with the development of acceptable scars and, above all, they avoid scars in the donor areas, such as grafts. However, the main obstacles to the application of dermal substitutes include the slow vascularization of the substitute and the risk of bacterial infection. Also in this case, various bioactive factors, such as exosomes, can play an important role in the angiogenesis process, just as they themselves can release antibiotics at the site of use with maintenance of the right bacterial biofilm. Nanomaterials have great potential for tissue engineering. The effective delivery of bioactive factors (including growth factors, peptides, and nucleic acids) by nanomaterials is of growing research interest [86]. Growth factors such as vascular endothelial growth factor, platelet-derived growth factor, and angiopontin are able to promote vascularization by promoting the formation of new blood vessels. However, these factors, in addition to being expensive, are very unstable and therefore must be preloaded to ensure an effective, continuous, and gradual release. Currently, sponge-like or film-like scaffolds composed of nanoparticles and nanospheres [87] have been developed, which are loaded with growth factors and therefore these are released into the wound site to promote neoangiogenesis. In view of the difficulty of using commercial growth factors, recent studies envisage a “personalized” application using the patient’s platelet-rich plasma in combination with different biomaterials in order to improve their properties to stimulate wound healing and regeneration of tissues [88]. Interestingly, the combination of polymers and platelet-rich plasma provides controlled spatiotemporal local release. Similarly, scaffolds composed of bioactive materials such as hydrotalcite [89] have been designed that are capable of loading antibiotics that can be released gradually and over time in the wound site, ensuring “sterility” against colonization [90,91]. The combination of biomaterials suitable for wound management (such as dermal substitutes) with fibroblasts, or dermal stem cells is the result of advances in tissue bioengineering. Historically, dermal substitutes can be used in combination with skin grafts or with skin micrografts. In this regard, the Meek technique offers an alternative method of covering large areas in the absence of adequate donor sites [92,93]. The aim of this technology is to mechanically disintegrate epidermal–dermal tissue, gathering autologous micrografts enriched in progenitor cells, growth factor, and extracellular matrix [94,95]. When combined with collagen sponges, micrografts can form a viable and proliferative bio-complex, enhancing their regenerative potential [96].

### 2.3. D Bioprinting Applications

The replacement and embellishment of patients’ tissues has largely been a dilemma that reconstructive surgeons have scuffled, but traditional surgical therapies frequently have restricted abilities. Conventional biomaterials, not infrequently, may outcome in postoperative complications such as contraction, infection, or rejection. The main complication of biomaterials, in current use, includes infection, which is emerging as a serious threat as bacteria often adhere to the surface of biomaterials (especially porous ones), are difficult to remove, and show high resistance to bactericides [97]. On the other hand, autologous tissue transplantation may develop the question of donor-site impairment. 3D bioprinting technology permits the planning of customized implants, introducing an elevated level of accuracy and shape. Moreover, implantations arranged with printed biomaterials and individual patient’s cells have greater biocompatibility and minor immunogenicity than traditional biomaterials. The current definition of 3D Bioprinting is based on the three-dimensional printing of cells or biomaterials on a specific substrate according to the requirements of the cellular morphology and microenvironment, tissue functionality, forming biologically functional three-dimensional constructs [98]. Three steps are involved in the 3D bioprinting work: pre-processing, processing and post-processing [99]. The use of 3D bioprinting generally incorporates many areas of tissue engineering, such as skin, bone, cartilage, and, in this regard, many areas of reconstructive surgery [100,101]. In this aspect, it must be taken into consideration that tissues contain different cells and different extracellular matrix, and therefore a universal printing material cannot be used for these tissues; therefore, the right material must be chosen for different tissues, on the features of biocompatibility and mechanical peculiarities [102]. Today, the principal bioprinting materials are: (i) inorganic materials, such as metals (titanium), bioceramics, clay, hydroxyapatite, graphene, carbon nanotubes [103,104] that have high strength, low elasticity, osteoconductivity, and corrosion resistance; (ii) synthetic polymers, such as polycaprolactone (PCL), polylactide (PLA) and polyurethane (PU) [105] that have variable degradation rate, good stiffness, high elasticity, and malleability; (iii) natural biopolymers, such as alginate, gelatin, collagen, fibrin, decellularized ECM, and hyaluronic acid [106,107] that have low cost, easy extraction, biodegradability, high biocompatibility, antibacterial properties, non-immune response, and provide a cell-specific microenvironment. In addition, the ingredients of the bioink can be “added” by including extracellular vesicles, exosomes, and growth factors that make the bioprinting functionalized material [108,109]. In plastic reconstructive surgery, these 3D bioprinting materials can be used in patients with severe burns, diabetic ulcers, tumors, or traumatic severe skin tissue defects, which currently can only be treated with alloplastic grafts or pedunculated or free autologous flaps. To avoid scarring in the donor site or discomfort for the patient, this technology can be a new tool available to the plastic surgeon. Some authors have already used this technology for the regeneration of skin defects, and have combined 3D bioprinting materials with mesenchymal stem cells [110,111,112]. This research has highlighted how this technology is able to heal wounds in vivo by generating collagen and improving cell proliferation. Since the microenvironment in which 3D bioprinted MSCs were used was nutritionally deficient, various bioactive substances were applied to improve the capabilities of MSCs, such as angiogenesis [113]. Such regenerative “smart dressing” provided an appropriate microenvironment to enhance MSC proliferation and differentiation but also accelerated anti-inflammatory activities to promote wound healing by increasing the expression of wound healing factors. 3D bioprinting technology, with or without bioactive substances, permits a personalized and tailored patient-needs and prevents the surgical complications and adverse reactions of traditional surgery [114]. When designing a scaffold or biomaterial, physiologically relevant design decisions must be made, depending on the target site and the function needed. The 3D bioprinting technology considers various characteristics, such as biological, physical, mechanical, and structural, for optimal scaffold design and manufacturing. The customization of scaffold characteristics is conceptualized based on the target tissue and required purpose. Biological issues are related to biocompatibility, biodegradability, and non-toxic properties; furthermore, structural and physical issues are related to porosity, mechanical behavior, pore size, and surface topography. Finally, chemical issues are related to capacity to include growth factors, proteins, drugs, and antibiotics in the biomaterial [115]. In addition, biomaterials, as well as being customizable and functionalized, can be sensitive to the stimuli of the microenvironment (such as temperature, pH, and infections) in which they are used. These biomaterials exploit nanotechnological properties as vehicles for the controlled release of drugs or growth factors that respond spatiotemporally to specific endogenous stimuli, promoting controlled tissue regeneration or the controlled management of infections [116]. The 3D bioprinting field is a rapidly expanding area of research, albeit with questions related to the correct mixture of cells, an adequate printable scaffold, and the ideal microenvironment to mimic native tissue. 3D bioprinting is still an embryonic technology, evidenced by the fact that most current studies are only in vitro proof of concept [117].

### 2.4. Exosomes

Exosomes (EVs) are extracellular vesicles involved in intercellular communication and in the transfer of cargo incorporating proteins, lipids, and nucleic acid [118,119]. Exosomes have been discovered in many bodily fluids [120], emphasizing their role in intercellular communication in both physiological and pathological proceeding. Currently, a correlation between exosomes and chronic pain has been demonstrated [121]. In reconstructive and plastic surgery, in particular in hand surgery, there are many situations, such as neuropathic pain from peripheral neuropathy, from nerve injury, from complex pain regional syndrome (CPRS), or chronic inflammatory pain from osteoarthritis in which there is currently no consolidated therapy. In this context, exosomes appear to be a possible therapeutic strategy by transferring substances that improve pain and have immunoprotective and anti-inflammatory potential [122,123]. Neuropathic pain is a type of chronic pain that occurs following an injury or disease in the peripheral nervous system [124], and appears to be related to neuroinflammation [125,126]. Various clinical studies have shown that exosomes can improve neuropathic pain by reducing proinflammatory cytokines, promoting vascular regeneration, promoting neuronal proliferation and function by axonal regrowth and Schwann cell activation [127,128], which contribute to providing a favorable microenvironment for peripheral nerve regeneration. Another pathology responsible for chronic inflammatory pain is osteoarthritis. In this pathology, the pain is classically considered to be nociceptive, resulting from the abnormal load of a damaged joint. Changes in joint biomechanics interact on nociceptive nerve endings by opening ion channels and generate this specific type of pain [129]. Exosomes from different sources, such as synovial fibroblasts, chondrocytes but also adipose tissue, plays a role in reducing excessive chondrocyte death by inhibiting apoptosis process [130]. Moreover, exosomes inhibit extracellular matrix degradation, resulting in alleviation of synovial inflammation. Degeneration of cartilage tissue during osteoarthritis is caused by the presence of chronic inflammation. ASCs have the potential to attenuate degenerative and inflammatory processes in OA, through EVs isolated from human ASCs exerting chondroprotective functions through multiple mechanisms, such as reducing the production of inflammatory mediators, decreasing the release of metalloproteinase activity, and improving the production of the anti-inflammatory cytokine [131,132].

## 3. Conclusions and Perspectives

Translational medicine has brought an evolution to classical research in the field of cell biology. Here, the models are based on conditions that, although on the one hand managing to explain biological events and processes, on the other hand failing to explain and translate such biological knowledge into clinical practice. Advances in biology and cellular engineering have led to the development of applied translational medicine, which allows us to examine biological processes and their interactions in a more biomimetic context and therefore allows us to understand how they function in a real microenvironment. Many medical disciplines are similar to this new translational model, and reconstructive plastic surgery is certainly one of them. Modern translational research has new technological approaches available, which can certainly be applied to the “reconstructive ladder”, making it translational, based on more sophisticated tissue-engineering models. Adipose tissue today has a wide application in the field of soft tissue reconstruction, but has limitations that can be resolved with the use of biomaterials that provide additional reinforcement or support, providing critical signals for regeneration. Further success in the use of these biomaterials will require a deeper understanding of the components of the ECM in regeneration and the role of extracellular vesicles in immunomodulatory processes. Defining the physical, mechanical, and biomechanical properties of biomaterials and their integration with the host, as well as defining degradation properties, is also an area under development. There is a continuing need for further interdisciplinary research to understand biological processes in order to create safe and durable translational technologies in the clinic. The “translational reconstructive ladder” is not just an algorithm that allows the plastic surgeon to correctly perform surgery. Today, reconstruction capabilities have been increased a thousand times with incredible resources and cutting-edge technological devices. All of this, if inserted within this “translational reconstructive scale”, allows us to act in a more precise, rapid, and also safer way, to better satisfy reconstructive and aesthetic needs. No one, alone, will be able to include within their own cultural experience all the tools available in the “translational reconstructive scale”, but the best reconstructive surgeon will somehow guarantee the best alternative.

The other side of the coin is understanding how the success of translational research from the laboratory to the bedside depends on the creation of a multidisciplinary and interdisciplinary scientific team capable of collaborating and communicating bidirectionally, so that all actors involved (academia, non-profit foundations, pharmaceutical and biotechnology companies, hospitals) synchronize efforts toward a common good. There is currently consensus that there is a rift involving the translatability of basic science into bedside applications. This gap, defined by many authors as the “valley of death” [133], has caused a slowdown in the development of translational medicine fundamentally linked to the high costs of production and the healthcare system, reproducibility, clinical relevance, and regulatory. Considering that reconstructive surgery is a procedure that restores the “form and above all function” of a tissue after damage or disease, plastic surgeons will certainly be the major users of the translational techniques, through an integrated approach between technologies coming from engineering, biomaterials science, cell biology, and reconstructive microsurgery.

## Data Availability

No new data were created or analyzed in this study. Data sharing is not applicable to this article.
